# Senegal’s Iodine Puzzle: Iodine Status, Salt Iodization, and Dietary Iodine Sources

**DOI:** 10.1016/j.cdnut.2025.106008

**Published:** 2025-03-24

**Authors:** Rita Wegmüller, Maguette F Beye, Ndeye F Ndiaye, Volkan Cakir, Ndèye Yaga Sy, Sitor P Ndoure, Maty D Camara, Malick Anne, Nafissatou B Lo, Jessica Rigutto, Germana H Leyna, Amid Abdelnour, Fabian Rohner, Nicolai Petry, James P Wirth, Valeria Galetti

**Affiliations:** 1 GroundWork, Fläsch, Switzerland; 2 Helen Keller International, Dakar, Senegal; 3 Institut de Technologie Alimentaire et Comité Sénégalaise pour la Fortification des Aliments en Micronutriment, Dakar, Senegal; 4 Direction de la Lutte contre la Maladie, Ministère de la Santé et de l'Action Sociale, Dakar, Senegal; 5 Conseil National de Développement de la Nutrition, Dakar, Senegal; 6 Laboratory of Nutrition and Metabolic Epigenetics, ETH Zurich, Switzerland; 7 Global Center for the Development of the Whole Child, University de Notre Dame, Notre Dame, IN, United States; 8 Tanzania Food and Nutrition Centre, Dar es Salaam, Tanzania; 9 Biolab, Amman, Jordan; 10 Direction de la Santé de la Mère et de l'Enfant, Ministère de la Santé et de l'Action Sociale, Senegal

**Keywords:** iodine, salt iodization, urinary iodine concentration, dietary iodine sources, nonpregnant females, processed foods, iodine apportioning, Senegal

## Abstract

**Background:**

Iodine is vital for human health, and its deficiency is linked to severe disorders. Although salt iodization is practiced in Senegal, evidence shows declining household iodized salt coverage.

**Objectives:**

This survey assessed iodine status in nonpregnant females and examined dietary sources contributing to their iodine intake.

**Methods:**

This cross-sectional survey was conducted in 2023 and was nationally representative. Using stratified sampling, data were collected from 866 households and from 657 nonpregnant females aged 15–49 y. Median urinary iodine concentration (UIC), urinary sodium concentration, and household salt iodine concentration were analyzed, with the apportioning of iodine sources through statistical methods to estimate iodine intake from native dietary sources, iodized salt in processed foods, and iodized household salt.

**Results:**

Iodine sufficiency was observed with a median UIC of 252 *μ*g/L, yet regional disparities exist, with some areas showing more than adequate iodine concentrations. Coverage with adequately iodized salt was low (19%), with higher availability in urban areas. Females in urban areas showed higher UICs than those in rural areas, which might be related to a higher contribution from adequately iodized salt. Processed foods, including bouillon, contributed about half to iodine intake, with household salt accounting for only a small portion (9% in urban and 5% in rural areas).

**Conclusions:**

Despite low iodized salt coverage at the household level, nonpregnant Senegalese females have adequate iodine intake due to iodine in processed foods. Effective monitoring of iodized salt used for processed foods is essential to mitigate potential excess intake whereas ensuring continued iodine sufficiency in all population groups.

## Introduction

Iodine is a critical micronutrient for human health, essential for thyroid hormone production and metabolic regulation. Iodine deficiency disorders can lead to a range of health issues, from goiter and developmental delays to increased mortality risk in severe cases. Universal salt iodization has been the cornerstone strategy to prevent iodine deficiency disorders globally [1].

In Senegal, salt iodization (SI) has been mandatory since 2001 [2], and iodine concentrations are defined to be between 20 and 60 ppm at the retail level according to the Economic Community of West African States standards that Senegal has adopted in 2015 [3]. Iodized salt coverage has increased since 2001; according to demographic and health surveys, there has been a small but steady increase in household coverage of iodized salt from 56% to 71% between 2010 and 2018 [4,5]. A salt coverage assessment conducted in 2014 even reported coverage with iodized salt at 81%, but only 37% of households used adequately iodized salt [6]. Reasons for this discrepant finding were repeatedly ascribed to the salt production landscape with many small-scale salt producers, quality control issues during production, and inconsistent supply of potassium iodate. Further, domestic funding would be important to strengthen the monitoring and enforcement of the legislation.

Iodine status among females of reproductive age at the national level was last assessed in 2014, and results reported were comparable to those in 2010 [4]: a borderline status at the national level [median urinary iodine concentration (UIC) 98 μg/L] but with a lower median UIC in females residing in rural areas indicating a risk of iodine deficiency there [7].

However, although UIC can provide region-specific estimates, it does not inform on the proportion of iodine that comes from iodized salt (either used in processed foods or from discretionary salt used in the household) or from native dietary iodine sources. Since other sources than iodized salt used in the household play an increasingly important role in modern societies, we used the apportioning method suggested by van der Haar et al. [8] to estimate where the iodine in females of reproductive age is mostly coming from. By including urinary sodium concentration (UNaC) from the same spot urine sample used to measure UIC, as well as household SI concentration, this statistical method allows the apportioning of the UIC to native dietary iodine, iodine from processed food salt, and iodine from household salt.

Results presented here are based on findings from the Senegal salt and sodium intake survey (SSIS) 2023, a nationally representative survey aiming at a broader scope around salt and sodium intake [9]; here, we aim to provide an update on the coverage with (adequately) iodized salt and iodine status in nonpregnant females and aim to estimate the proportion of iodine coming from different dietary sources.

## Methods

### Study design and participants

The basic design of the SSIS was that of a national cross-sectional stratified survey based on a probability sample with 5 strata and a 3-staged sampling approach. The 5 strata were West (Dakar, Thiès), Center (Fatick, Kaffrine, Diourbel, Kaolack), North (Saint-Louis, Louga, Matam), East (Tambacounda, Kédougou), and South (Sédhiou, Ziguinchor, Kolda). In the first stage of sampling, 75 enumeration areas (EA) – 15 per stratum – were randomly selected with probability proportional to size. The second stage of sampling consisted of random selection with an equal probability of 12 households in each EA. The selection of households in the EA was made after conducting a listing of all households residing in the EA. In the third sampling stage, 1 eligible woman 15–59 y of age was selected from each household with equal probability.

The survey protocol was approved by the Comité Nationale d’Ethique de la Recherche pour la Santé (SEN22/130). The study protocol can be accessed at https://osf.io/h9dqw/.

### Procedures

The module on household-level questions was administered first upon oral consent from the household head or any other qualified adult respondent. This module collected information on household composition, including the age and sex of household members and meals consumed outside the home, as well as details on the household head, water, and sanitation. It also gathered data on land and animal ownership, dwelling characteristics, and food purchase patterns for salt and other salt contributors usually consumed in small quantities like bouillon, mustard, and seasoning sauces. The household daily consumption of these small-quantity food items was calculated by the amount usually purchased by the household divided by the time such quantity usually lasts. The individual daily consumption was calculated by partitioning the household daily consumption among its family members using the adult male equivalent method [10,11] and then corrected by the frequency each family member usually eats out of the house. At the end of the household questionnaire, a salt sample was collected.

Before administering individual interviews, written informed consent was sought from the selected woman. Individual questionnaire modules included topics such as age, marital status, educational level, a 24-h recall targeted to high-salt foods consumed in larger quantities (e.g., bread, chips, processed meat, fish, and seafood), and a food frequency section for composite dishes that were consumed out of the house. To estimate portion size for the food items asked about in the 24-h recall module a picture catalog was prepared. The French version of the questionnaires can be found at https://osf.io/h9dqw/.

Upon completion of the individual questionnaire, urine samples were collected. All females collected a spot urine sample (for a small sub-sample, this was collected as part of a 24-h urine sample used to estimate sodium intake in the main survey [9]) into a prelabeled small urine container. Urine samples were divided into aliquots and stored in cold boxes until transportation to a regional health facility within 24 h of sample collection, where they were stored at –20°C until shipment for analysis.

UNaC was analyzed by Biolab (Amman, Jordan) using a Roche c503 SER clinical analyzer. The Tanzania Food and Nutrition Centre (Dar es Salaam, Tanzania) measured iodine concentration in household salt samples using iodometric titration as well as iodine concentration in spot urine samples from all females between 15 y and 49 y of age (the target population for iodine status assessment) using a modified Sandell-Kolthoff reaction according to Pino et al. [12]. Both laboratories regularly participate in external quality control schemes, showing highly satisfying laboratory performance and conducting daily quality control during the analyses of samples.

In addition to household and individual data, vendor information was collected from 1 randomly selected restaurant/food stall located in or near each of the 75 selected clusters. A short questionnaire was administered to assess the relative contribution of salt and bouillon in the composite dishes prepared on the day of the interview using the proportional piling method[13,14].

### Statistical analysis

Data analysis was done using STATA version 18. Data analysis included the calculation of proportions of households using iodized salt and the calculation of medians as measures of central tendency for iodine concentration in salt and urine. Overall proportions and medians were calculated using weighted analysis to account for the unequal probability of selection in the 5 strata. The statistical precision of all estimates was assessed using 95% confidence limits. All measures of precision, including confidence limits and χ^2^ *P* values for differences, were calculated accounting for the complex cluster and stratified sampling used by the SSIS. The results on females’ iodine status are presented as weighted median UIC and weighted 95% confidence intervals (CIs) calculated by bootstrapping for complex survey data using STATA’s “bsweights” command [15]. In addition, UIC, UNaC, SI, wealth index, and bouillon consumption are also presented as weighted mean and SD or weighted median and IQR, depending on whether the distribution was normal or not normal, respectively.

After applying appropriate sampling weights, we ran a multiple generalized linear regression with natural log-transformed UIC data as the dependent variable and UNaC, SI, type of residence, stratum, wealth index, and bouillon consumption as explanatory variables. We calculated UIC portion estimates for native iodine, processed food salt iodine, and household salt iodine by using the intercept and regression coefficient as per van der Haar et al. [8].

## Results

### Response rates and demographic characteristics

Overall, 881 households were randomly selected to participate in the survey, of which 866 (98%) completed the household interview. The reasons for nonparticipation were mainly the absence of a competent respondent and refusal. Of the households selected, 60% were in rural areas, and 67% had a male household head. Ninety-three percent of households drank safe water, but only 54% had adequate sanitation, and 62% were food insecure. Of the 813 eligible nonpregnant females between 15 and 59 y of age within these households, 743 (91%) completed the interview and provided a urine sample, and 657 of those were between 15 and 49 y of age and thus included for iodine status assessment. Two UIC results were excluded from the analysis as they were identified as upper outliers. The mean age of the 655 nonpregnant females with UIC was 29.1 y, 506 were nonlactating (79%), and 149 were lactating. Slightly over 70% were married, and 43% never attended school.

### Household coverage of iodized salt

Among the households included, 763 (88%) reported having salt at home at the time of the survey. One household did not want to provide a salt sample, and 5 households had insufficient quantities of salt for any analysis. Salt iodine results were available from 755 households. Of these households, 45% reported that their salt was iodized, 12% reported noniodized salt, and 43% said that they didn’t know whether the salt was iodized or not. Of all the salt samples collected at the households, 65.1% (95% CI: 58.2, 71.4) were not iodized (<5 ppm), 16.3% (95% CI: 12.8, 20.6) were insufficiently iodized, and 18.6% (95% CI: 13.1, 25.7) were adequately iodized (≥15 ppm). Table 1 shows that twice as many households use adequately iodized salt in urban (26.7%) than in rural (13.3%) areas (*P* = 0.053); subsequently, there are also stratum-specific differences with the lowest coverage in the Center (4.6%) and the highest coverage in the West (33.1%). The proportion of iodized salt was higher among the fine salt samples (25.2%) than among the coarse salt samples (11.5%). Around 60% of salt samples were not stored in their original packaging (due to open purchases; data not shown), and the proportion of adequately iodized salt was similar to those samples originating from a package with a label stating that the salt was iodized (around 20% of samples).

**TABLE 1:** Proportion of households using adequately iodized salt.

Characteristic	*n*	Median iodine concentration (ppm)	Adequately iodized (≥15 ppm)		
			%	95% CI	*P*
**Residence**					0.053
Urban	229	4.2	26.7	(16.3, 40.5)	
Rural	526	3.2	13.3	(7.6, 22.2)	
**Stratum**^1^					0.023
East	160	3.2	16.9	(10.2, 26.5)	
North	159	2.1	15.7	(7.0, 31.7)	
Center	152	2.1	4.6	(1.0, 18.8)	
West	136	5.3	33.1	(21.4, 47.2)	
South	148	3.2	14.9	(6.0, 32.3)	
**Wealth quintile**^2^					0.171
Lowest	200	3.2	13.5	(6.2, 27.1)	
Second	209	2.1	11.5	(6.5, 19.8)	
Middle	131	3.7	22.2	(11.3, 39.0)	
Fourth	113	3.2	17.4	(8.6, 31.9)	
Highest	102	4.2	28.5	(15.9, 45.6)	
**Household food security**^3^					0.439
Secure	257	3.2	18.7	(11.8, 28.2)	
Mildly insecure	60	3.2	9.7	(3.9, 22.1)	
Moderately insecure	212	3.2	21.1	(14.1, 30.5)	
Severely insecure	226	3.2	19.3	(11.1, 31.5)	
**Packaging of salt**					0.842
Original packaging stating salt is iodized	192	3.7	21.6	(13.3, 33.2)	
Original packaging, no mention of iodization	116	3.2	16.7	(9.3, 28.2)	
Undetermined, not in the original package	334	3.2	17.8	(11.0, 27.4)	
Undetermined for other reasons	85	3.2	19.8	(10.1, 35.1)	
**Salt type**					0.017
Fine	333	4.2	25.2	(16.3, 36.9)	
Coarse	413	2.1	11.5	(6.4, 19.9)	
**Total**	**755**	**3.2**	**18.6**	**(13.1, 25.7)**	

Abbreviation: CI, confidence interval.

1 Strata is defined as follows: East (Tambacounda, Kédougou), North (Saint-Louis, Louga, Matam), Center (Fatick, Kaffrine, Diourbel, Kaolack), West (Dakar, Thiès), and South (Sédhiou, Ziguinchor, Kolda).

2 Assessed by the Demographic and Health Surveys Wealth Index.

3 Assessed by the Food and Nutrition Technical Assistance Household Food Insecurity Access Scale.

### Iodine status of nonpregnant females of reproductive age

The median UIC of 252 *μ*g/L indicates overall iodine sufficiency (Table 2). However, females residing in urban areas have higher UIC (280 *μ*g/L) than females from rural areas (230 *μ*g/L), and there are differences between the different strata. Median UIC is lowest in the East stratum with 187 *μ*g/L. Interestingly, median UIC is highest in the West and the Center stratum (∼270 *μ*g/L), despite the Center stratum showing the lowest coverage of adequately iodized salt, whereas the West stratum has the highest coverage. Further, median UIC increases with increasing wealth and with increasing household SI category. Females from households using adequately iodized salt have a median UIC of 353 *μ*g/L, indicating excess iodine intake. However, females from households using noniodized salt still have a median UIC of 229 *μ*g/L, suggesting important dietary iodine intake from sources other than iodized salt used at the household level. The median UIC in the 149 nonpregnant lactating females (235 *μ*g/L) is comparable with the median in the 506 nonpregnant nonlactating females (257 *μ*g/L).

**TABLE 2:** Median urinary iodine concentration in nonpregnant females 15–49 years of age.

Characteristic	*n*	Median UIC (*μ*g/L)	95% CI	*P*
**Age group (in years)**				0.308
15–19	134	268	(235, 300)	
20–29	219	239	(194, 283)	
30–39	177	240	(198, 282)	
40–49	125	258	(224, 292)	
**Residence**				0.002
Urban	189	280	(217, 344)	
Rural	466	230	(207, 253)	
**Stratum**^1^				<0.001
East	138	187	(162, 212)	
North	143	262	(232, 291)	
Center	139	269	(235, 304)	
West	99	272	(225, 319)	
South	136	231	(196, 266)	
**Wealth quintile**				0.004
Lowest	178	196	(162, 229)	
Second	172	250	(125, 374)	
Middle	123	254	(138, 371)	
Fourth	101	277	(208, 346)	
Highest	81	280	(185, 376)	
**Educational level**				0.203
Never attended school	302	237	(195, 279)	
Attended school	353	263	(241, 284)	
**Currently breastfeeding**				0.102
No	506	257	(229, 285)	
Yes	149	235	(195, 275)	
**Household salt iodization**				<0.001
None (<5 ppm)	398	229	(206, 252)	
Insufficient (5–14.9 ppm)	103	263	(224, 302)	
Adequate (≥15 ppm)	92	353	(212, 494)	
**Total**	**655**	**252**	**(231, 274)**	

Abbreviations: CI, confidence interval; UIC, urinary iodine concentration.

1 Strata is defined as follows: East (Tambacounda, Kédougou), North (Saint-Louis, Louga, Matam), Center (Fatick, Kaffrine, Diourbel, Kaolack), West (Dakar, Thiès), and South (Sédhiou, Ziguinchor, Kolda).

**FIGURE 1:**
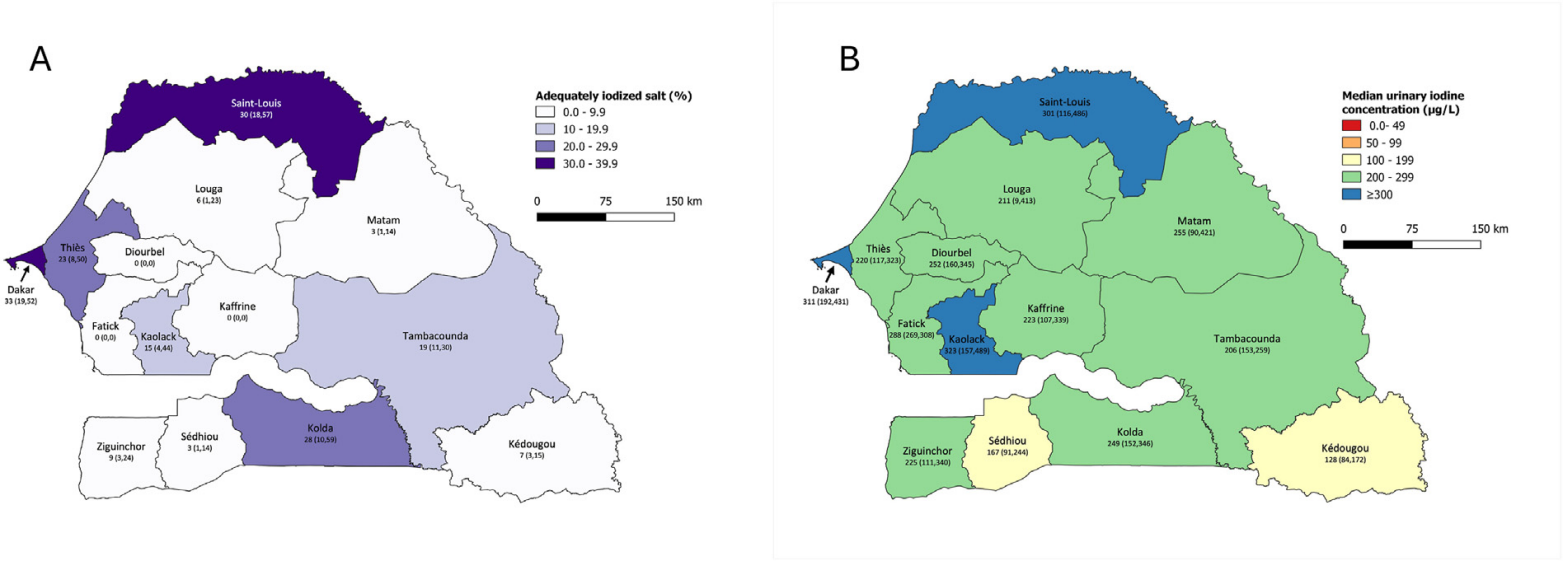
Geographic distribution of (A) households using adequately iodized salt (≥ 15 ppm) and (B) median urinary iodine concentration in nonpregnant females 15–49 y of age. Values for each region are percentage (95% CI) in panel (A) and median UIC (95% CI) in panel (B). CI, confidence interval; UIC, urinary iodine concentration.

The 2 maps in Figure 1 highlight some regional differences in household SI and UIC. The regions of Dakar and Saint-Louis show the highest coverage of adequately iodized salt, albeit still only at 30–40% (Figure 1A). In 8 of the 14 regions, household coverage with adequately iodized salt is below 10%. The geographic distribution of median UIC in nonpregnant females (Figure 1B) highlights that in all regions, iodine intake is at least adequate, but in 3 regions, females are at risk of excess intake (Dakar, Saint-Louis, and Kaolack). In 2 of these 3 regions, SI is highest with 30–40%. On the contrary, median UIC indicates iodine sufficiency even in the 8 regions with coverage of adequately iodized salt below 10%.

### Apportioning of UIC to different dietary sources of iodine

**FIGURE 2:**
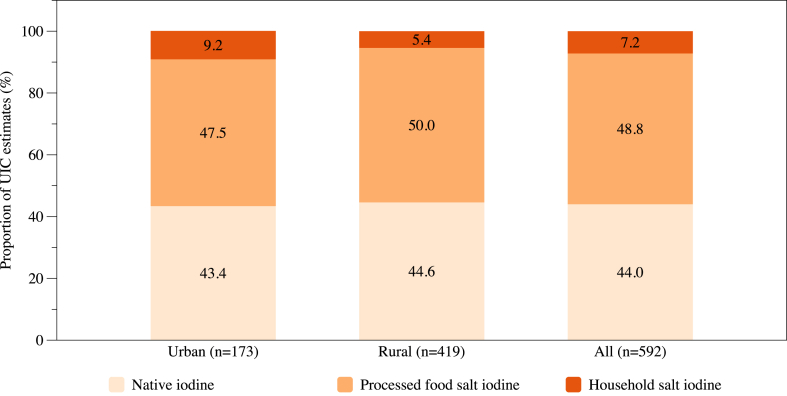
Proportion of UIC estimates in nonpregnant females 15–49 y of age by type of residence and overall. UIC, urinary iodine concentration.

Complete data for UIC, UNaC, and SI was available for 592 nonpregnant females (Supplemental Table 1). UNaC, SI, and wealth quintile are associated with UIC in a multiple regression model, whereas type of residence, stratum, and bouillon consumption are not (Supplemental Table 2). Considering that urban and rural settings might differ in terms of dietary origin of iodine (because median UIC is higher in urban than rural areas, and SI tends to be higher in urban areas) and that wealth quintile and type of residence were highly correlated in a bivariate model, we decided to calculate the apportioning of UIC to the different dietary iodine sources overall, but also by type of residence. Overall, urban and rural-specific UIC portions that correspond to the different sources of iodine in the diet of nonpregnant females in Senegal are reported in Table 3. In urban areas (*n* = 173), we estimate native iodine to contribute 132 *μ*g/L to overall dietary iodine intake, which is above the 100 *μ*g/L minimum threshold for iodine sufficiency, whereas the contribution in rural areas (*n* = 419) is at that threshold with 97 *μ*g/L. Thus, coverage through other sources is more important in rural than urban areas, where it may even lead to excess intake. The contribution of iodine from salt used in processed foods is estimated to be slightly higher than that from native iodine: 145 *μ*g/L in urban and 109 *μ*g/L in rural areas. However, the contribution from iodized salt used in the household is small, with 28 *μ*g/L and 12 *μ*g/L in urban and rural areas, respectively. Around half of the UIC contribution is from processed food salt (50.0% in rural and 47.5% in urban areas; Figure 2). The contribution from household salt is below 8% overall and is smaller in rural areas, at 5.4%, than in urban areas, with 9.2%.

**TABLE 3:** Urinary iodine concentration portions estimates (*μ*g/L; geometric mean, 95% CI) in nonpregnant females 15–49 y by type of residence.

	Urban (*n* = 173)		Rural (*n* = 419)		All (*n* = 592)	
Native iodine	132	(129, 136)	97	(94, 100)	111	(106, 116)
Processed food salt iodine	145	(125, 165)	109	(96, 121)	123	(111, 135)
Household salt iodine	28	(14, 42)	12	(7, 17)	18	(12, 24)
Total UIC	305	(285, 325)	217	(201, 233)	251	(235, 268)

Abbreviations: CI, confidence interval; UIC, urinary iodine concentration.

## Discussion

This nationally representative survey demonstrated adequate iodine status in nonpregnant females with a risk of excess iodine intake in some areas despite a low coverage of adequately iodized household salt.

The coverage of households with iodized salt has been declining in Senegal in the past years. Although coverage with iodized salt increased from 56% in 2010 [4] to 81% in 2014 [6], it started declining to 71% in 2018 [5] and 35% (in this survey) in 2023. Similarly, the proportion of households using adequately iodized salt declined from 48% in 2010 to 37% in 2014 and was only 19% in 2023. Median household salt iodine concentration was only 3.2 (IQR: 1.6–7.4) ppm in this survey compared to a median concentration of 15 ppm in 2010. The findings of all surveys are consistent in that more households in urban areas use iodized salt at home than in rural areas and that coverage increases with increasing wealth.

In view of this low coverage of (adequately) iodized salt at the household level, one would anticipate low iodine status in the population. However, our data on UIC in nonpregnant females has shown adequate or above adequate iodine status in all regions in Senegal, even in those with a very low coverage of iodized household salt. Data on UIC are rather scarce, with 2 national surveys in 2010 and 2014 showing borderline iodine status in nonpregnant females with a median UIC of 92 *μ*g/L and 98 *μ*g/L, respectively, despite a rather high household coverage of iodized salt [4,7]. In contrast, a decade later, the median UIC in nonpregnant females in our survey was 252 *μ*g/L, with more than half of the regions showing a coverage of iodized salt below 10%. These findings suggest that iodine has been delivered through alternative sources, such as iodized salt from processed foods and condiments. A study modeling iodine intake revealed that bouillon is an important source of dietary iodine in Senegal [16]. In this study conducted in 2014, different types of bouillon cubes and powders were analyzed for their iodine content, and results showed a wide range of iodine content between 1.2–20.0 *μ*g iodine per gram of bouillon, which was attributed to the use of iodized salt to produce the bouillon products. The authors further calculated that the salt used to produce the bouillon products was iodized at a concentration of 2–33 ppm. These results suggest that some bouillon producers used iodized salt, whereas others did not. The inclusion of iodized salt in processed foods was recommended by the WHO in 2014 in order to achieve optimal iodine nutrition for the population [17], and the use of iodized salt in processed foods is an industrial standards requirement in the Economic Community of West African States[3], a standard that Senegal adopted in June 2015. More recent data on iodine content in bouillon products in Senegal is not available, but it would be important to estimate how bouillon and other processed foods contribute to iodine intake.

Comparable to our findings, a study conducted in 2014 in northern Ghana has shown adequate iodine status with a median UIC of 242 *μ*g/L in school children with 72% of households not using iodized salt [18]. However, the median (IQR) iodine content in bouillon cubes was 31.8 (26.8–43.7) *μ*g/g, which is higher than that found in the study in Senegal in the same year [16]. In both studies, the variation in iodine content between the different brands was large. In Ghana, the estimated intake of iodine from bouillon was 88 *μ*g/d, contributing to around two-thirds of the daily recommended iodine intake in children [19], whereas the contribution from drinking water and milk-based products was negligible [18]. Bouillon consumption is also important in Senegal. The SSIS has shown that 94% of households in Senegal purchase bouillon cubes or powders without any differences between socioeconomic groups [9], which could make bouillon an important contributor to iodine intake if iodized salt is used for their production. The mean daily bouillon consumption per adult household member at home was 2.9 g, which is comparable to the 2.8 g and higher than the 1.9 g estimated in 2 national surveys in Senegal in 2014 and 2018 [7,20]. This does not consider bouillon consumed as part of composite dishes out of the house. Even though only 12% of adults indicated that they regularly consumed meals out of the house [9], the contribution of iodine from these meals might not be negligible. A survey among 92 restaurants and small food stalls in the whole country has shown that 95% of the composite dishes prepared on the day of the survey used both salt and bouillon [9]. Considering all of this, and assuming that the iodine content of bouillon was at around 25 ppm as stated on the packages of one of the most used brands, a daily bouillon consumption of around 3 g would lead to a daily intake of around 75 *μ*g, which would considerably contribute to iodine intake.

To further understand our findings, we used the apportioning method to statistically apportion the UIC in the nonpregnant female population to iodine from native dietary sources, from iodized salt used in processed foods, and from iodized salt used at the household level [8]. The original study used data from 3 population-representative national iodine nutrition surveys of nonpregnant females in Kenya, India, and Senegal. The Senegal data was from the survey in 2014 [7], and it was estimated that 61% of the iodine came from native iodine sources, 26% from processed foods containing iodized salt, and 13% were from iodized salt used at the household level [8]. The proportion of iodized household salt was lower in Senegal than in Kenya and India (both 17%), but as our data shows, this proportion decreased further to 7% in 2023. In contrast, the proportion of processed food salt became most important at 49%, and the proportion of native iodine sources declined to 44%. This is in line with the discrepancy between the low household coverage of (adequately) iodized salt and adequate (or above adequate) iodine status in nonpregnant females. On the contrary, salt used in processed foods, with the main contributor likely being bouillon, seems important and suggests that iodized salt is used during production of such foods or iodine is added as a fortificant to such foods containing salt during production. Some producers indicate the use of iodized salt on the ingredients list, whereas others do not specify this. There is currently no legislation to enforce the declaration of the use of iodized salt on ingredient lists. The variation in iodine content might be wide as the SSIS has shown that >10 different bouillon brands were being used at the household level [9]. However, 93% of the households mentioned 1 of the 2 leading brands (69% and 24% coverage, respectively) as the main brand consumed by the household. Both brands claim to use iodized salt and, thus, are likely contributing most to iodine intake. However, the 49% of iodine estimated to come from processed food salt is unlikely to only originate from bouillon as, in that case, the salt used for bouillon production would have had to be iodized at around 100 ppm, or alternatively, iodine was additionally added during production. Bread, sandwiches, and savory snacks contributed to ∼15% of salt intake in Senegal [9], which could also contribute to iodine intake if iodized salt is used for their production. Processed foods containing salt and, therefore, foods potentially contributing to iodine intake identified in earlier studies are bread, instant noodles, dried fish, meat products, potato chips, and biscuits [[21], [22], [23]]. However, the only African country included was Egypt. It will, therefore, be important to investigate the consumption of processed food sources and their iodine content in Senegal.

Native iodine sources were estimated to contribute almost half to overall iodine intake. The estimated urinary iodine portion of 111 *μ*g/L would translate to an intake of ∼102 *μ*g iodine per day, assuming that 92% of ingested iodine is excreted in urine [24], which would cover two-thirds of the recommended intake for an adult [25]. Potential native iodine sources include seafood, dairy products, and water. However, water and dairy products were found to be negligible sources in Ghana [18], and locally grown staples and vegetables are unlikely to contain higher iodine concentrations as there is no indication of a higher use of fertilizers between 2016 and 2022 in general and the most commonly used fertilizers do not contain any iodine [26]. Further investigation of native iodine food sources and possible regional differences would be important.

This study provides a detailed assessment using a nationally representative sample and a stratified sampling method, enhancing the generalizability and accuracy of the iodine status of nonpregnant females of reproductive age in Senegal. By integrating UIC, UNaC, and SI, the apportioning method provides a multifaceted view of iodine nutrition, and although allowing a nuanced analysis of iodine sources, it offers valuable insights for refining targeted nutritional interventions, overcoming the limitations of assessing the impact of universal SI solely through household SI. However, this approach relies on assumptions about the proportionate contribution of dietary sources to both urinary iodine and sodium, which might not always hold. The correction for native-source sodium would have allowed more accurate estimates for processed food salt UIC portions. However, salt intake is high in Senegal [9], and native sodium from foods is likely to contribute little to overall sodium intake.

In conclusion, nonpregnant females of reproductive age in Senegal show adequate iodine status with a risk of excess iodine intake in a few regions. This contrasts with the low coverage of iodized household salt. Iodized household salt contributes very little to iodine intake, whereas iodine from processed food salt is the most important source. Senegal is a country with many small-scale salt producers, which makes universal SI difficult. Salt from small-scale producers is often targeted at household consumption, and if it is not exported or used by the food industry to produce processed foods, iodization concentrations may not be that critical in this context. However, large-scale producers that are likely providing salt to the food industry, as well as exporting salt, need to make sure their salt is adequately iodized and monitored. It is important to continue monitoring the use of adequately iodized salt in processed food production and the iodine status of the population to avoid a risk of excess intake. Particularly, populations living in areas where adequately iodized salt from large-scale producers is available might be at risk of excess intake.

## Author contributions

The authors’ responsibilities were as follows – RW, MFB, VC, JR, FR, NP: designed research; RW, MFB, NFN, NYS, SPN, MDC, MA, NBL, GHL, AA, JPW: conducted research; JPW, VG: analyzed data; RW: wrote paper; RW, FR, VG: had primary responsibility for final content; and all authors: read and approved the final manuscript.

## Data availability

Deidentified data described in the manuscript, code book, and analytic code will be made available upon request pending, application and approval.

## Funding

This research was funded by the Bill and Melinda Gates Foundation, grant number INV-007916. The funders were involved during the early stages of study conceptualization but had no role in the actual design of the study, in the collection, analyses, or interpretation of data, in the writing of the manuscript, or in the decision to publish the results.

## Conflict of interest

Volkan Cakir reports financial support was provided by Bill & Melinda Gates Foundation. All other authors report no conflicts of interest.
