# Supplementary material for: Senegal’s Iodine Puzzle: Iodine Status, Salt Iodization, and Dietary Iodine Sources
Source: Curr Dev Nutr. 2025 Mar 24;9(5):106008. doi: 10.1016/j.cdnut.2025.106008 (PMC12059682; doi:10.1016/j.cdnut.2025.106008)
Supplement: multimedia component 1 [file mmc1.pdf]

Supplementary data

Supplemental Table 1: Descriptive characteristics of non-pregnant women 15-49 years of age with complete UIC, UNaC and SI data by type of residence

	N	UIC (µg/L)	UNaC (mmol/L)	SI (ppm)	Wealth Index	Bouillon consumption (g/day)
Urban	173	294 (209, 499)	179 ± 67	4.2 (2.1, 14.3)	3.3 ± 1.5	2.5 (1.5, 3.9)
Rural	419	226 (132, 341)	181 ± 85	3.2 (1.1, 5.3)	-1.1 ± 2.7	2.5 (1.7, 3.6)
All	592	252 (156, 382)	180 ± 78	3.2 (1.6, 7.4)	0.6 ± 3.1	2.5 (1.6, 3.7)

Values are median (IQR) or mean ± SD. UIC, urinary iodine concentration; UNaC, urinary sodium concentration; SI, salt iodization

Supplemental Table 2: Association of UIC with different explanatory variables in non-pregnant women 15-49 years of age

Explanatory factors	Estimate	95%CI	t-statistic	p
Intercept	4.6985	4.3778, 5.0193	29.2	<0.001
UNaC	0.0039	0.0028, 0.0049	7.3	<0.001
SI	0.0077	0.0023, 0.0132	2.8	0.006
Residence (ref. urban)				
Rural	-0.1459	-0.3787, 0.0868	-1.3	0.215
Stratum (ref. East)				
North	0.0870	-0.2161, 0.3902	0.6	0.569
Center	0.2306	-0.0442, 0.5054	1.7	0.099
West	0.0176	-0.2825, 0.3178	0.1	0.907
South	-0.0279	-0.2870, 0.2312	-0.2	0.830
Wealth index	0.0526	0.0150, 0.0901	2.8	0.007
Bouillon consumption	0.0002	-0.0330, 0.0334	0.0	0.990

UIC, urinary iodine concentration; UNaC, urinary sodium concentration; SI, salt iodization. Weighted slope estimates are β coefficients from generalised linear regression with natural log-transformed UIC as dependent variable.
